# Editorial: Early phase clinical trials for the development of novel immunotherapeutic anti-cancer agents

**DOI:** 10.3389/fimmu.2025.1750018

**Published:** 2025-12-01

**Authors:** Agnese Losurdo, Anastasia Lalioti, Angelo Dipasquale, Matteo Simonelli

**Affiliations:** 1 Department of Biomedical Sciences, Humanitas University, Pieve Emanuele, Milan, Italy; 2 Medical Oncology and Hematology Unit, IRCCS Humanitas Research Hospital, Rozzano, Milan, Italy

**Keywords:** immunotherapy, early phase clinical trials, solid tumors, biomarkers, immune-related adverse events (IRAEs)

Over recent years, immunotherapy has reshaped oncological treatment, shifting focus from direct cytotoxicity to immune modulation. Despite their transformative impact, monoclonal antibodies targeting immune checkpoints induce durable remissions in approximately 20-30% of patients, depending on the tumor type (1), with the exception of melanoma, with long term follow-up from CheckMate 047 trial indicating a potential for cure even in the metastatic setting (2, 3). Building on this premise, and with the goal of overcoming the current limitations of immune checkpoint inhibitors (ICIs), several combinatorial approaches, together with novel emerging immunotherapeutic strategies such as bispecific antibodies, adoptive cell therapy (e.g.: tumor infiltrating lymphocytes (TILs) therapy, chimeric antigen receptor (CAR)-T cell/T cell receptor (TCR)-transduced T cell therapy) and vaccination strategies, have now entered clinical development, with some already transitioning into clinical practice.

Specifically, CAR-T cell therapy in solid tumors faces intrinsic and extrinsic cancer hurdles: immunosuppressive elements of the tumor microenvironment (TME), trafficking and infiltration complexities, preventing CAR-T cells from effectively infiltrating tumors, and tumor heterogeneity, with mechanisms like antigen loss, allowing tumor cells to evade immune detection (4). In this complex context, Funasaka et al. presented a phase I trial evaluating a CAR-T cell product targeting Ephrin type-B receptor 4 (EPHB4), a molecule broadly expressed in solid tumors, including soft tissue neoplasms. This platform derives from a modified ephrin B2 ligand, enabling tumor specific recognition while minimizing T cell exhaustion and preserving cytotoxicity mediated killing. Even though such strong biological background sounds promising, challenges remain in mitigating on-target/off-tumor toxicities, ensuring long-term persistence and preventing secondary malignancies.

Key constraints to take into account when designing trials for immunomodulating agents are (a) the potential identification of reliable biomarkers to guide patient selection and optimize treatment strategies, (b) the early detection and mitigation of immune-related adverse events (irAEs) and (c) the choice of the optimal timing for maximizing immunotherapeutic strategies potential.

The heterogeneity of tumor-host crosstalk renders a universal biomarker approach unrealistic. Currently, predictive biomarkers such as programmed cell death ligand 1 (PD-L1) expression, tumor mutational burden (TMB) and mismatch repair deficiency (dMMR) are routinely implemented. However, their performance remains inconsistent, often serving better as stratification tools rather than definitive predictors of response. For this reason, there is a growing potential of minimally invasive biomarkers, including circulating tumor DNA (ctDNA) and serum factors to provide early indicators of therapeutic benefit. Jia et al. presented a focus on predictive biomarkers for the therapeutic combination of autologous natural killer (NK) cells with PD-1 inhibitor (Sintilimab) in non-small cell lung cancer (NSCLC) patients who had progressed to first-line platinum-based chemotherapy. A significant association was observed between treatment efficacy and both clearance of ctDNA and higher levels of tumor infiltrating PD-L1 + NK cells following treatment, suggesting that they may serve as prognostic markers.

On the safety side, although ICIs related toxicities, which might potentially affect every organ and tissue, are well noted, accurate prediction tools remain lacking. This is even more true with immunomodulating agents other than ICIs, such as recombinant pro-inflammatory cytokines, which are now attracting renewed interest in the field of immune-oncology, in monotherapy and in combination therapy with other immunomodulatory drugs (5). For instance, Interleukin 2 (IL2) has long been investigated as a target cytokine promoting T cell expansion, and has been approved for the treatment of metastatic melanoma and renal cell carcinoma; however, its wide use has been hampered by its toxic profile, with frequent grade 3 and 4 adverse effects (6). In this context, Aguirrechu et al. conducted a phase I study testing an engineered IL2 protein with an increased affinity for CD8 and NK cells and reduced stimulation of regulatory T cells. As a result, a favorable safety profile was reported, dose-limiting toxicity was not reached and IL2 related severe adverse events (SAEs) (e.g.: vascular leak syndrome and multiorgan dysfunction) were not detected, with most frequent toxicities being the well-known symptomatic set composed of chills, fever, and tachycardia.

Lastly, when testing immunomodulating agents, we need to consider tumor biology to set the best timing of administration; indeed, neoadjuvant immunotherapy has been proven to improve surgical results and overall outcomes, across different tumor types, given the presence of the intact full tumor antigenic repertoire (7). In this background lay both studies presented by two different research groups in esophageal cancer. The clinical trial of Zhou et al. evaluated whether perioperative treatment with the anti-PD-1 antibody tislelizumab, in combination with four cycles of neoadjuvant chemotherapy, followed by post-operative adjuvant PD-1 blockade, could improve pathological complete response (pCR) in patients with locally advanced esophageal cancer. The study reported a pCR of 44% and disease-free survival (DFS) of 75% after 1 year of follow up. Notably, 16% of the participants enrolled, did not conclude preoperative treatment, either due to disease progression or unacceptable toxicity. Most treatment-related adverse events were mild to moderate in severity, including neutropenia, nephrotoxicity, cardiovascular and gastro-intestinal complications. Overall, the study demonstrated encouraging clinical activity supporting further evaluation of this perioperative combination in larger studies. Similarly, the phase Ib/II clinical trial of Zhang et al. investigated a neoadjuvant regimen that combines chemotherapy with the PD-1 inhibitor tislelizumab as well as the addition of low dose radiation treatment in patients with locally advanced esophageal cancer. The rationale stems from the ability of radiation treatment to reshape tumor microenvironment, creating inflamed and immune-responsive cancer lesions. While this triple-modality approach holds promise for synergistic efficacy, optimal radiation dose needs to be defined, as well as management of overlapping side effects. Defining this therapeutic balance will be fundamental to ensure improved outcome now offset by treatment related adverse effects.

Collectively, these studies illustrate the multifaced evolution of cancer immunotherapy, emphasizing the importance of integrating immune components to enhance checkpoint blockade and fueling immune response. While innovative monotherapy and combination treatment regimens improve survival, their efficacy remains limited by toxicity risks. Central to these advances is biomarker development, as optimizing patient selection in preclinical trials improves efficacy and mitigates adverse events. All together such trials reflect a shift towards precision-guided targeted treatment, poised to redefine the next generation of cancer therapy.
